# The lateral habenula is critically involved in histamine-induced itch sensation

**DOI:** 10.1186/s13041-020-00660-y

**Published:** 2020-08-27

**Authors:** Hyoung-Gon Ko

**Affiliations:** 1 Department of Anatomy and Neurobiology, School of Dentistry, Kyungpook National University, 2177 Dalgubeol-daero, Daegu, 41940 South Korea; 2 Department of Biological Sciences, College of Natural Sciences, Seoul National University, 1 Gwanangno, Gwanak-gu, Seoul, 08826 South Korea

## Abstract

Lateral habenula (LHb) is a brain region acting as a hub mediating aversive response against noxious, stressful stimuli. Growing evidences indicated that LHb modulates aminergic activities to induce avoidance behavior against nociceptive stimuli. Given overlapped neural circuitry transmitting pain and itch information, it is likely that LHb have a role in processing itch information. Here, we examined whether LHb is involved in itchy response induced by histamine. We found that histamine injection enhances Fos (+) cells in posterior portion within parvocellular and central subnuclei of the medial division (LHbM) of the LHb. Moreover, chemogenetic suppression of LHbM reduced scratching behavior induced by histamine injection. These results suggest that LHb is required for processing itch information to induce histaminergic itchy response.

## Main text

Various external stimuli coming through the periphery are transmitted to the brain, and the brain processes them for survival of higher organism. Among stimuli, pruritogens usually evoke negative sensation and induce scratching behavior to reduce it. Primary sensory neurons expressing pruriceptors such as histamine and PAR2 receptor transmit pruriceptive stimuli to second-order neurons in spinal cord [1, 2]. Growing studies have revealed the neural circuitry conveying pruriceptive information in the spinal cord [3, 4]. Although there are specific interneurons activated only by pruritogen, it has been accepted that nociceptive and pruriceptive information are usually transmitted common spinal neural circuit [3, 5]. After leaving spinal cord, itch information is conveyed to several brain regions via spinothalamic tract like as nociception transmission. In contrast to spinal level, although a few studies reported that some brain regions are activated by pruriceptive stimuli [6–8], it is largely unknown how and which brain regions process itch information.

As mentioned previously, neural circuitry transmitting itch information largely overlaps with that of pain information. Thus, it is conceivable that brain regions involving pain processing also mediate itch information. Among brain regions engaging the processing of pain information, the lateral habenula (LHb) is known to be activated by aversive stimuli and induces avoidance behavior [9, 10]. Many studies have reported that the LHb in rodent and human is activated by noxious stimuli [11–15]. The lateral hypothalamic region (LHA) is directly innervated by sensory stimuli transmitted via spinothalamic tract, and directly projects to LHb. In addition, it was reported that spinal projection neurons also directly project to the LHb in cat [16]. When the LHb receives sensory information with negative valence, the LHb mediates avoidance behaviors by manipulating aminergic circuits [17, 18]. Given the role of LHb and aversive property of itchy sensation, it is reasonable that the LHb is involved in the processing of itchy information. Although a few studies showed that LHb is activated by pruritogens [6], there is no direct evidence that LHb is involved in the processing of itchy information. Here, we aimed to reveal whether the LHb is required for the processing of itchy sensation.

**Fig. 1:**
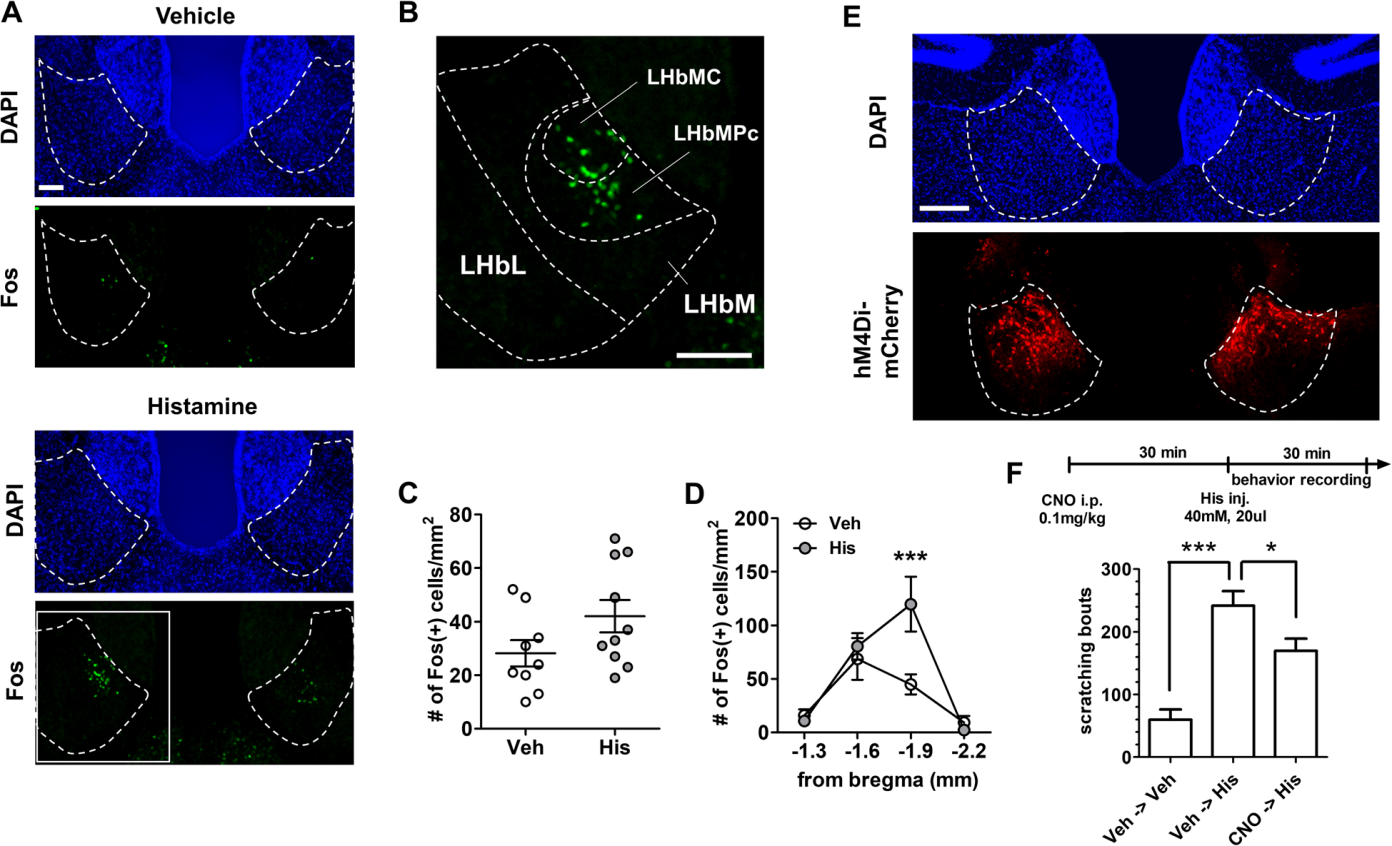
Histamine-induced itchy responses mediated by LHb. **a** Representative images of Fos expressing LHb after vehicle or histamine injection. Areas surrounded white dotted lines indicate LHb. Brain slices were counterstained with DAPI to identify LHb region. Scale bar, 200 μm. **b** High magnified images of rectangular region in Fig. 1b. It is noted that, in histamine-injected group, Fos (+) cells were concentrated into the parvocellular (LHbMPc) and central (LHbMC) subnuclei within the medial division of the LHb (LHbM). Scale bar, 200 μm. **c** Histamine injection tends to increase Fos (+) cells in the overall LHb (*n* = 9 ~ 10/group; unpaired t-test; t_17_ = 1.885, *p* = 0.0767). Veh; Vehicle, His; Histamine. **d** Histamine-induced Fos expression was significantly increased only in posterior part of LHb (*n* = 9 ~ 10/group, two-way ANOVA followed by Bonferroni posttest: effect of drug, F_(1, 68)_ = 4.001, *p* < 0.05; effect of slice, F_(3, 68)_ = 18.35, *p* < 0.005, effect of interaction, F_(3, 68)_ = 4.232, *p* < 0.01; posttest, * *p* < 0.005). **e** Representative images showing hM4Di-mCherry expression in LHb after the end of behavioral experiments. Areas surrounded white dotted lines indicate LHb. Brain slices were counterstained with DAPI to identify LHb region. Scale bar, 200 μm. **f** Upper: experimental scheme for testing the role of LHb for itchy information processing. Lower: Histamine-induced scratching responses were inhibited by suppression of LHb activities. (*n* = 9 ~ 10/ group, one-way ANOVA followed by Tukey’s multiple comparison test; F _(2, 26)_=22.43, *p* < 0.001; * *P* < 0.05, *** *p* < 0.001)

To investigate whether the LHb is activated by itch stimulus, we used histamine as pruritogen. Ninety minutes after intradermal injection of 40 mM histamine (20ul, in saline, His) solution into the rostral part of the back, mice were decapitated and brains were processed by the procedure of immunohistochemistry. Based on the fact that immediate-early gene, c-fos expression reflect neuronal activation, we examined Fos-immunoreactivities in the LHb. When we analyzed overall LHb, although His injection tended to increase Fos (+) cells, it was not significantly different compared to vehicle-injected group (Fig. 1a and b). However, we observed that His injection robustly increased Fos (+) cells only in restricted area of LHb (Fig. 1c) and this increase is mainly concentrated into posterior part of the parvocellular (LHbMPc) and central (LHbMC) subnuclei within medial division of the LHb (LHbM) [19]. These results suggested that pruriceptive stimulus activates neurons located in the limited area of LHb.

Given the results showing His-induced activation of LHb, we sought to examine whether LHb is required for itchy response induced by His injection. To suppress neuronal activities of LHb, we used inhibitory GiDREADD system. The hM4Di-mcherry, delivered by AAVs, was expressed under CaMKII promoter based on that major cell type of LHb is glutamatergic [9]. Clozapine-N-oxide (CNO) (0.1 mg/kg) was intraperitoneally injected to suppress neuronal activities expressing hM4Di. His injection robustly increased scratching response compared to vehicle injection group, but pretreatment of CNO reduced scratching responses (Fig. 1d and e). These results showed that neuronal activities in LHb contribute to itchy behavioral response.

In this study, we found that histamine injection activated neurons located in restricted area of LHbMPc and LHbMC. Also, we revealed that LHb activation is required for histamine-induced scratching behavior. Actually, the LHb is consist of several distinct subnuclei divided based on the characteristics of habenular cells [19]. Although the connectivity between each subnuclei of LHb and other brain regions has not revealed in detail, it was revealed that LHbM region including LHbMPc and LHbMC receives afferent input originated from midbrain and limbic area such as hypothalamus and basal forebrain [9]. Among these brain regions, LHA is well known to be activated by pruritogens [6]. Thus, it seems that LHA innervates a part of LHbMC and LHbMPc after histamine injection. Also, in our preliminary experiments, we observed that histamine activated paraventricular hypothalamus (PVN) (data is not shown), which is known to project to LHbM regions [20]. These two inputs to LHbM are known to involved in aversion and escape behaviors [20–24]. Thus, it is likely that histamine stimulates LHbMC and LHbMPc via LHA and/or PVN. But, it is still unclear why only a small part, posterior area, of LHbMC and LHbMPc was specifically activated by histamine injection.

In contrast to afferents of LHb, efferents involving itch information processing are not obscure. The LHb suppress dopaminergic neurons in ventral tegmental area (VTA) via GABAergic interneurons within VTA or in rostromedial tegmental nucleus (RMTg) [18, 25]. Moreover, pruritus-induced scratching behavior was initiated by activation of GABAergic neuron in VTA, and dopaminergic neuron was activated immediately after scratching behavior [25]. Given the connectivity between LHbM and VTA, it seems that LHbM modulates VTA activities for processing itchy information.

To determine specifically whether LHbMC and LHbMPc subnuclei mediate the processing of itchy information, further study showing specific features of each subnuclei, such as molecular markers, is required. Fortunately, recent studies began to report that transcriptome is not uniformly distributed, show spatial distribution pattern in LHb [26]. Identification of molecular makers specifically expressed in LHbMC and LHbMPc will help to reveal the function of these subnuclei on itchy information processing. In addition, although we used histamine as a pruritogen in our study, many studies indicated that different types of pruritogens stimulate distinct neurons and activate partially overlapped brain areas [27–29]. Thus, it is worth to examine whether nonhistaminergic pruritogens are also processed by same LHb region activated by histamine. Finally, our study will help to understand how itch stimuli is processed by the brain, and contribute to develop treatment for pathological itch such as atopic dermatitis.

## Supplementary information

**Additional file 1.**

## Data Availability

All data generated or analysed during this study are included in this published article [and its supplementary information files].

## References

[1] Liu T, Ji R-R. New insights into the mechanisms of itch: are pain and itch controlled by distinct mechanisms? Pflugers Arch. 2013;465(12) [cited 2020 Jul 5]. Available from: https://www.ncbi.nlm.nih.gov/pmc/articles/PMC3796138/. 10.1007/s00424-013-1284-2 PMC3796138 23636773

[2] Min H, Lee H, Lim H, Jang YH, Chung SJ, Lee CJ (2014). TLR4 enhances histamine-mediated pruritus by potentiating TRPV1 activity. Mol Brain.

[3] Akiyama T, Carstens E, Carstens E, Akiyama T (2014). Spinal Coding of Itch and Pain. Itch: Mechanisms and Treatment [Internet].

[4] Zhang L, Jiang G-Y, Song N-J, Huang Y, Chen J-Y, Wang Q-X (2014). Extracellular signal-regulated kinase (ERK) activation is required for itch sensation in the spinal cord. Mol Brain.

[5] Han L, Ma C, Liu Q, Weng H-J, Cui Y, Tang Z (2013). A subpopulation of nociceptors specifically linked to itch. Nat Neurosci.

[6] Jeong K-Y, Kang J-H (2015). Investigation of the pruritus-induced functional activity in the rat brain using manganese-enhanced MRI. J Magn Reson Imaging.

[7] Zhang T-T, Shen F-Y, Ma L-Q, Wen W, Wang B, Peng Y-Z (2016). Potentiation of synaptic transmission in rat anterior cingulate cortex by chronic itch. Mol Brain.

[8] Koga K, Yamada A, Song Q, Li X-H, Chen Q-Y, Liu R-H (2020). Ascending noradrenergic excitation from the locus coeruleus to the anterior cingulate cortex. Mol Brain.

[9] Hu H, Cui Y, Yang Y (2020). Circuits and functions of the lateral habenula in health and in disease. Nat Rev Neurosci.

[10] Shelton L, Becerra L, Borsook D (2012). Unmasking the mysteries of the habenula in pain and analgesia. Prog Neurobiol.

[11] Shelton L, Pendse G, Maleki N, Moulton EA, Lebel A, Becerra L (2012). Mapping pain activation and connectivity of the human habenula. J Neurophysiol.

[12] Lehner M, Taracha E, Skórzewska A, Wisłowska A, Zienowicz M, Maciejak P (2004). Sensitivity to pain and c-Fos expression in brain structures in rats. Neurosci Lett.

[13] Gao DM, Hoffman D, Benabid AL (1996). Simultaneous recording of spontaneous activities and nociceptive responses from neurons in the pars Compacta of Substantia Nigra and in the lateral Habenula. Eur J Neurosci.

[14] Benabid AL, Jeaugey L (1989). Cells of the rat lateral habenula respond to high-threshold somatosensory inputs. Neurosci Lett.

[15] Li Y, Wang Y, Xuan C, Li Y, Piao L, Li J, et al. Role of the Lateral Habenula in Pain-Associated Depression. Front Behav Neurosci. 2017;11 [cited 2020 Jul 28]. Available from: http://journal.frontiersin.org/article/10.3389/fnbeh.2017.00031/full. 10.3389/fnbeh.2017.00031 PMC5318408 28270756

[16] Craig AD (2003). Distribution of trigeminothalamic and spinothalamic lamina I terminations in the cat. Somatosens Motor Res.

[17] Stamatakis AM, Stuber GD (2012). Activation of lateral habenula inputs to the ventral midbrain promotes behavioral avoidance. Nat Neurosci.

[18] Jhou TC, Fields HL, Baxter MG, Saper CB, Holland PC (2009). The Rostromedial tegmental nucleus (RMTg), a GABAergic afferent to midbrain dopamine neurons, encodes aversive stimuli and inhibits motor responses. Neuron.

[19] Zahm DS, Root DH (2017). Review of the cytology and connections of the lateral habenula, an avatar of adaptive behaving. Pharmacol Biochem Behav.

[20] Zhang L, Hernández VS, Vázquez-Juárez E, Chay FK, Barrio RA. Thirst Is Associated with Suppression of Habenula Output and Active Stress Coping: Is there a Role for a Non-canonical Vasopressin-Glutamate Pathway? Front Neural Circuits. 2016;10 [cited 2020 Jul 17]. Available from: https://www.frontiersin.org/articles/10.3389/fncir.2016.00013/full. 10.3389/fncir.2016.00013 PMC4814529 27065810

[21] Zhang G-W, Shen L, Zhong W, Xiong Y, Zhang LI, Tao HW (2018). Transforming Sensory Cues into Aversive Emotion via Septal-Habenular Pathway. Neuron.

[22] Stamatakis AM, Swieten MV, Basiri ML, Blair GA, Kantak P, Stuber GD (2016). Lateral hypothalamic area Glutamatergic neurons and their projections to the lateral Habenula regulate feeding and reward. J Neurosci.

[23] Lecca S, Meye FJ, Trusel M, Tchenio A, Harris J, Schwarz MK (2017). Aversive stimuli drive hypothalamus-to-habenula excitation to promote escape behavior. Elife.

[24] Lazaridis I, Tzortzi O, Weglage M, Märtin A, Xuan Y, Parent M (2019). A hypothalamus-habenula circuit controls aversion. Mol Psychiatry.

[25] Su X-Y, Chen M, Yuan Y, Li Y, Guo S-S, Luo H-Q (2019). Central Processing of Itch in the Midbrain Reward Center. Neuron.

[26] Hashikawa Y, Hashikawa K, Rossi MA, Basiri ML, Liu Y, Johnston NL (2020). Transcriptional and Spatial Resolution of Cell Types in the Mammalian Habenula. Neuron.

[27] Papoiu ADP, Coghill RC, Kraft RA, Wang H, Yosipovitch G (2012). A tale of two itches. Common features and notable differences in brain activation evoked by cowhage and histamine induced itch. NeuroImage.

[28] Papoiu ADP, Kraft RA, Coghill RC, Yosipovitch G (2015). Butorphanol suppression of histamine itch is mediated by nucleus Accumbens and Septal nuclei. J Invest Dermatol.

[29] Davidson S, Zhang X, Yoon CH, Khasabov SG, Simone DA, Giesler GJ (2007). The itch-producing agents histamine and Cowhage activate separate populations of primate Spinothalamic tract neurons. J Neurosci.

