# Supplementary material for: The lateral habenula is critically involved in histamine-induced itch sensation
Source: Mol Brain. 2020 Aug 27;13:117. doi: 10.1186/s13041-020-00660-y (PMC7457247; doi:10.1186/s13041-020-00660-y)
Supplement: Supplementary file 1 — Additional file 1. [file 13041_2020_660_MOESM1_ESM.docx]

Jul. 20th, 2020

**The lateral habenula is required for histamine-induced itch sensation**

Hyoung-Gon Ko^1,2,*^

^1^Department of Anatomy and Neurobiology, School of Dentistry, Kyungpook National University, 2177 Dalgubeol-daero, Daegu 41940, South Korea

^2^Department of Biological Sciences, College of Natural Sciences, Seoul National University, 1 Gwanangno, Gwanak-gu, Seoul 08826, South Korea

*Correspondence: hgko@knu.ac.kr

**This file includes:**

**Materials and methods**

**References**

**Materials and Methods**

***Animals***

Male C57BL/6NCrljBgi mice aged between 6 and 8 weeks were purchased from Samtako Bio Korea. Animals were housed in standard laboratory cages on a 12-hour light-dark cycle with access to food and water *ad libitum*. All the experiments were approved by the Institute of Laboratory Animal Resources of Seoul National University (SNU-180409-3).

**Immunohistochemistry**

Immunohistochemistry was performed essentially as described previously (1). Mice were anesthetized and transcardially perfused with PBS (0.1 M phosphate buffer, pH 7.4) followed by 4% paraformaldehyde (PFA) in PBS. Brains were collected and immersion-fixed with 4% PFA at 4°C overnight, cryo-protected with 30% sucrose in PBS for two days at 4°C, embedded in an OCT (optimum cutting temperature) compound (Tissue-Tek, Sakura Finetek). Coronal sections (40 μm thick) were made using a cryostat, and every 4th section was collected in 50% Glycerol in PBS. Sections were washed with PBS for 5 min three times, incubated with a blocking solution (PBS containing 10% normal goat serum and 0.3% Triton X-100) for 30 min at room temperature, and incubated with rabbit anti-c-Fos antibody (1:250, Santacruz) diluted in the blocking solution for two days at 4°C. Sections were washed with PBS containing 0.3% Triton X-100 (PBST) for 10 min four times, incubated with fluorescent-conjugated secondary antibodies (1:500) diluted in the blocking solution for 2 hours at room temperature, washed with PBST for 10 min four times. After sections were incubated with DAPI (0.2 μg/ml, Invitrogen) in PBS for 10 min at room temperature, sections were transferred onto glass slides and mounted with VECTASHIELD (Vector Laboratories). The sections were imaged with a fluorescent microscope, and the images were analyzed using ImageJ software.

**Behavior experiments after AAV injection**

AAV injection was performed essentially as described previously (2). AAV (serotype 1) expressing hM4Di-mCherry under CaMKII promoter was used to express inhibitory DREADD. AAVs (1 × 10^12^ gc/ml) were injected bilaterally into the LHb (AP -0.7 mm, ML ±0.5 mm, DV −3.0 mm, 0.2 μl/side) by stereotaxic surgery (Stoelting Co.). Two weeks after the stereotaxic surgery, mice were habituated to experimental apparatus during 30 min per day over 2 days. After last habituation, mice were briefly anesthetized with isoflurane and shaved in the rostral part of the back with an electrical clipper. Clozapine-N-oxide (CNO) (0.1 mg/kg) or saline as a vehicle was intraperitoneally injected 30 min before itch test. To deliver pruritogen, mice were briefly anesthetized with isoflurane and intradermally injected with histamine (20 μl, 40 mM in saline, Sigma-Aldrich) using a Hamilton syringe. Mice were transferred into plexiglass chamber and their behavior was videotaped for 30 min at the top of chamber after awaking from anesthesia. The number of scratching bouts using hind paw was manually counted by blind experimenter. After behavioral experiments, mice were decapitated to confirm the expression of inhibitory DREADD. Brains were collected and processed in the same way as immunohistochemistry by omitting blocking and immunoreaction steps.

***Data analysis***

Statistical comparisons were made using the unpaired t-test, one-way ANOVA (Tukey test was used for post-hoc comparison) and two-way ANOVA (Bonferroni posttest was used for post-hoc comparison). Data were presented as the mean ± S.E.M. In all cases, p < 0.05 was considered statistically significant.

**References**

1. Ko H-G, Choi J-H, Park DI, Kang SJ, Lim C-S, Sim S-E, et al. Rapid Turnover of Cortical NCAM1 Regulates Synaptic Reorganization after Peripheral Nerve Injury. Cell Rep. 2018 16;22(3):748–59.

2. Kang SJ, Kwak C, Lee J, Sim S-E, Shim J, Choi T, et al. Bidirectional modulation of hyperalgesia via the specific control of excitatory and inhibitory neuronal activity in the ACC. Mol Brain. 2015 Dec 2;8(1):81.
